# Health Equity and Access to COVID-19 Treatments Available through Emergency Use Authorizations

**DOI:** 10.1007/s40615-024-02094-x

**Published:** 2024-07-22

**Authors:** Candon Johnson, Carolyn Wolff, Jing Xu, On behalf of the N3C consortium

**Affiliations:** 1 Food and Drug Administration, Office of the Commissioner, Silver Spring, MD USA; 2 Federal Trade Commission, Bureau of Economics, Washington, DC USA; 3 Food and Drug Administration, Center for Drug Evaluation and Research, Silver Spring, MD USA

**Keywords:** COVID-19, Health Equity, Racial Health Disparities, Emergency Use Authorizations, 62P25

## Abstract

Understanding and evaluating equity in access to care is a critical component to ensuring health equity for all individuals. During the COVID-19 pandemic, the U.S. Food and Drug Administration made unprecedented use of its regulatory authority by authorizing the use of unapproved products through Emergency Use Authorizations (EUAs). We use data from the U.S. National COVID Cohort Collaborative (N3C) to understand how access to therapeutic products authorized under EUAs has varied across COVID-19 patients and over time. We find that Black patients were more likely to receive early EUA drugs while White patients were more likely to receive monoclonal antibodies. Male patients were more likely to receive any EUA drug than Female patients. Patients in Metropolitan areas were more likely to receive EUA drugs than patients in other regions. Additionally, differences in the rates of exposure to EUA drugs by gender, rural-urban classification, and length of stay decreased over time while differences by race and ethnicity have generally persisted. Our project identifies inequities in the rate of access to EUA drugs across patient groups that can inform policy makers in future planning and decision making.

## Introduction

In order to achieve health equity for all individuals, we need a better understanding of the heterogeneous impacts of health policies across diverse populations. Understanding how access to health care may vary across the United States is a critical component of this goal. Prior to the COVID-19 pandemic, the overall trend in access to care was improving for minority and disadvantaged groups, although significant disparities by race, ethnicity, household income, and location of residence remained [1]. While it is yet to be determined how the overall COVID-19 pandemic may have impacted these general trends, Alcendor (2020) finds that communities of color and other underserved populations living with limited access to social services have been particularly hard hit by the pandemic and continue to be among the most vulnerable [2].

A key public health measure used during the COVID-19 pandemic was the authorization of medical products under FDA’s Emergency Use Authorization (EUA) authority. While FDA made unprecedented use of this authority during the pandemic to help expedite the availability of medical products and strengthen the public health protections,^3^ it is currently unknown what impact it has had on health equity in the United States. In an effort to shed light in this area, we examine the health equity associated with access to drugs authorized under the FDA EUA authority during the COVID-19 pandemic.

The EUA authority allows FDA to authorize unapproved medical products or unapproved uses of previously approved medical products to be used in an emergency. On February 4, 2020, the Secretary of Health and Human Services (HHS) determined that there was a public health emergency involving the SARS-CoV-2 virus, which causes the COVID-19 illness. That determination provided the basis for justifying the authorization of emergency use of medical products during the COVID-19 pandemic [4]. FDA has since used the EUA authority to authorize numerous vaccines, therapeutic products, and medical devices to combat the COVID-19 pandemic.

By analyzing how access to drugs authorized under the EUA authority for COVID-19 has varied across diverse patient populations, our work contributes to the broader, expanding literature on health equity. Previous studies in this area have tended to focus on differences across race and ethnicity using a convenience sample of patients or examining a subset of the drugs authorized for COVID-19 [5,6]. Our study expands the literature in three ways. First, we use data from the National COVID Cohort Collaborate (N3C), the largest open U.S. database of patient electronic health records representing more than 15 million patients observed between January 2020 and September 2022 [7]. Second, we include in our analysis all drugs ever authorized under an EUA for COVID-19. Third, we seek to identify the presence and size of any possible differences in the use of these drugs for COVID-19 patients across a variety of demographic factors such as race, ethnicity, age, gender, and rural-urban classification, including how these differences changed over time throughout the pandemic. Our findings suggest disparities in EUA use during the COVID-19 pandemic and highlight the need for future research to incorporate a comprehensive set of controls for confounding factors to further understand the causes of the disparities shown.

## Data and Methodology

**Fig. 1:**
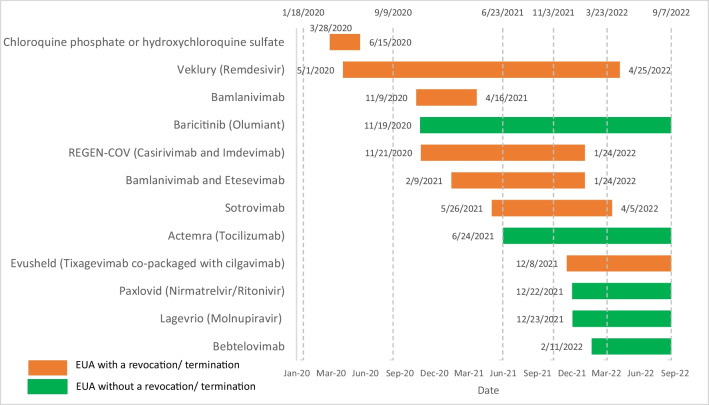
Drugs Authorized under an EUA for COVID-19

We first use the FDA’s website to identify drugs authorized for COVID-19 under an EUA as of September 2022. The website maintains lists of drugs with current EUAs and terminated or revoked EUAs [8]. Using these lists, we collect the drug name, the date of first EUA issuance, and the date of termination or revocation of the EUA if applicable. Each drug is listed in Fig. 1 with the date of first EUA issuance and the date of termination or revocation if applicable.^1^

We use data from the N3C to identify patients who received EUA drugs authorized for COVID-19 [7]. The N3C is the largest collection of COVID-19 data from electronic health records of patients at participating institutions in the United States who were tested for COVID-19 or who had symptoms related to COVID-19. Patients are not recruited for the N3C. Instead, participating sites, many of which are part of colleges and universities, provide data from the electronic health records of patients who were tested for COVID-19 or who had related symptoms. At the time of our analysis in September 2022, there were 76 participating sites releasing electronic health records data into N3C. The data include information on patient demographics, medical history, lab results, medications, and treatments. We limit the data to patients who tested positive for COVID-19 and compared the use of COVID-19 EUA drugs by race, ethnicity, gender, age, and rural-urban classification.

Our analysis of the N3C data begins with a sample of over 15 million patients observed between January 2020 and September 2022. Restricting the data to only COVID-positive patients reduces our sample to 6 million patients. Further restricting the data to those with full demographic information results in an analysis sample of 4.4 million patients.

The N3C includes data on patient race and ethnicity, gender, age, and geographic region. We separate race and ethnicity into two discrete variables. The variable for race has categories for White, Black, Asian, and Other Race, while the ethnicity variable is a binary indicator for Hispanic origin. We utilize Year of Birth to capture the age of the patient at the time of the study. Gender is categorized into Male and Female. To capture rural-urban classification, we utilize Rural-Urban Commuting Area Codes to create categories for Metropolitan (urbanized area with population of 50,000 or more), Micropolitan (urban cluster with a population of 10,000 to 49,999), and Small Town/Rural (urban cluster with a population of 2,500 to 9,999 or outside of an urbanized area or urban cluster) [9]. We also construct a variable for the length of stay. Using columns for Visit Start Date and Visit End Date, we categorize patients into Single Day and Multi-day Stays.

A single patient may have multiple visits recorded in the N3C data. We assume that the demographic variables for a single patient would not vary across visits. Therefore, when considering patient race and ethnicity, gender, age at the time of the study, and geographic region, we conduct our analysis at the patient level and consider a patient to be exposed to an EUA drug if they received an EUA drug during any visit. We conduct the analysis for length of stay, however, at the visit level, resulting in a greater number of total observations. At the visit level, we consider a patient to be exposed to an EUA drug if they received an EUA drug during that particular visit.

Table 1 summarizes the demographics of our sample of COVID-positive patients and presents them alongside those of the U.S. population during the same time period [10]. The average patient was born in 1979. Female patients represent 55.21% of the sample. A majority of patients are White (74.96%) and 87.09% are Not Hispanic or Latino. Most patients reside in metropolitan areas (83.15%), while 6.61% reside in small town/rural areas. A large portion (70.39%) of patient visits occur in a single day.

**Table 1:** Demographic Summary Statistics

Demographic	Number COVID-19 patients	Percentage of COVID-19 Patients	Percentage of U.S. Population
Gender			
Male	1,985,679	44.79%	49.24%
Female	2,447,704	55.21%	50.76%
Race			
White	3,080,392	74.96%	70.42%
Black	668,946	16.28%	12.62%
Asian	97,784	2.38%	5.64%
Other, including Multiple, Race	262,168	6.38%	11.32%
Ethnicity			
Not Hispanic or Latino	3,559,440	87.09%	81.82%
Hispanic or Latino	527,663	12.91%	18.18%
Rural Urban Commuting Area			
Metropolitan	3,686,442	83.15%	83.64%
Micropolitan	454,110	10.24%	8.93%
Small Town/Rural	292,831	6.61%	7.43%
Multi-day Stay			
Yes	1,619,974	29.61%	
No	3,850,929	70.39%	
Total Number of Patients	4,433,383	100%	

Data on gender, race, and ethnicity for the U.S. population are obtained from the 2020 American Community Survey, https://data.census.gov/cedsci. Data on Rural Urban Commuting Area for the U.S. Population are obtained from the 2010 Rural-Urban Commuting Area Code, https://www.ers.usda.gov/data-products/rural-urban-commuting-area-codes.aspx

We take a total difference approach and define a disparity as the total difference in the outcome measure between groups. Our primary approach to measuring this disparity is to calculate mean rates of exposure to EUA drugs by race, ethnicity, gender, age, geographic region, and length of stay and compare across groups. In addition to calculating rates of exposure to any EUA drugs, we also consider rates of exposure to two subsets of EUA drugs. The first subset, early EUA drugs, includes only the earliest drugs authorized for use against COVID-19, Remdesivir and Hydroxychloroquine. We consider these early EUA drugs separately because minority populations and people of color were particularly hard hit by the early stages of the pandemic. The second subset of EUA drugs that we consider separately includes all monoclonal antibodies (mAbs) authorized for use against COVID-19. Unlike earlier EUA drugs for COVID-19, mAbs were the first that became available largely on an outpatient basis, resulting in different availability and access patterns than the earlier drugs. Finally, we explore exposure to EUA drugs over waves of the pandemic. From January 18, 2020 to September 7, 2022, we use each local minimum in COVID-19 case data to identify the beginning of a wave and the subsequent local minimum to identify the end of a wave. Using this approach, we identify five distinct waves of the COVID-19 pandemic: Wave 1 from January 18, 2020 to September 9, 2020; Wave 2 from September 10, 2020 to June 23, 2021; Wave 3 from June 24, 2021 to November 3, 2021; Wave 4 from November 4, 2021 to March 23, 2022; Wave 5 from March 24, 2022 to September 7, 2022.

## Results

In Table 2, we report results for exposure to any EUA in column (1), exposure to early EUAs in column (2), and exposure to mAbs in column (3) by demographic group. We observe that 5.31% (320,102 out of 4,433,383) of COVID-positive patients received any EUA drug, 2.69% (119,214 out of 4,433,383) received an early EUA drug, and 2.05% (90,751 out of 4,433,383) received a monoclonal antibody EUA. The average year of birth for EUA recipients is approximately 1962. Early EUA recipients were older on average, and patients receiving monoclonal antibodies were about 3 years younger. Black patients had the highest exposure to any EUA drug and Asian patients had the highest exposure to early EUA drugs. White patients had the highest exposure to monoclonal antibodies. Non-Hispanic or Latino patients had more exposure to any EUA drug and monoclonal antibodies compared to Hispanic and Latino patients. Metropolitan areas received the most exposure to any EUA drug and early EUA drugs, while patients in Small Town/Rural areas had the lowest exposure to monoclonal antibodies. Unsurprisingly, patients that had a multi-day stay were much more likely to be exposed to any EUA drug and early EUA drugs. However, the difference between multi-day stay and single day stay narrowed for monoclonal antibodies.

**Table 2:** Mean EUA Exposure

Demographic	COVID-19 Patients received any EUA Drug	COVID-19 Patients received Early EUA Drug	COVID-19 Patients received EUA mAbs
Age Group (Average YOB)	1962	1959	1965
Gender			
Male	5.61% ***	3.11% ***	2.00% ***
Female	5.06% ***	2.35% ***	2.09% ***
Race			
White	5.34% *	2.32% ***	2.34% ***
Black	6.03% ***	3.82% ***	1.80% ***
Asian	5.64% ***	3.92% ***	1.10% ***
Other Race	1.96% ***	1.52% ***	0.38% ***
Ethnicity			
Hispanic or Latino	4.53% ***	3.32% ***	1.01% ***
Not Hispanic or Latino	5.71% ***	2.70% ***	2.35%
Rural Urban Commuting Area			
Metropolitan	5.52% ***	2.80% ***	2.15% ***
Micropolitan	4.27% ***	2.03% ***	1.77% ***
Small Town/Rural	4.25% ***	2.36% ***	1.20% ***
Multi-day Stay			
Yes	9.89% ***	7.03% ***	2.44% ***
No	4.99% ***	2.56% ***	1.87% ***
Overall	5.31%	2.69%	2.05%

The overall sample size is 4,433,383 patients

^*^p<0.1, **p<0.05, ***p<0.01

We also explore various EUA exposure measures by race across sex, geographic region, and length of stay. Table 3 reports these results. Patients of Other Race received the lowest exposure to any EUA drug, early EUA drugs, and mAbs across every demographic. Among Male patients, Asians experienced the highest rate of exposure to any EUA drugs and to early EUA drugs while Whites experienced the highest rate of exposure to mAbs. Black Male patients experienced higher rates of exposure than White Male patients to any EUA drug and to early EUA drugs, but lower rates of exposure to mAbs. Among Female patients, Blacks experienced the highest rate of exposure to any EUA drug and early EUA drugs, while Whites experienced the highest rate of exposure to mAbs. Overall differences in exposure to any EUA drug in metropolitan areas were smaller than in micropolitan and small town/rural areas, but the metropolitan areas experienced differences in rates of exposure to early EUA drugs across race that were similar to other regions. The findings by gender that White patients experienced the highest rate of exposure to mAbs seem to be driven by patients in Metropolitan areas. In Micropolitan and Small Town/Rural areas, Blacks and Asians had the highest rates of exposure to mAbs, respectively. Looking at EUA exposure during multi-day stays by race, White patients were less likely to be exposed to any EUA drug and early EUA drugs than Black and Asian patients; however, White patients had the highest rate of exposure to monoclonal antibodies.

**Table 3:** Mean EUA Exposure by Race

Demographics	COVID-19 Patients received an EUA Drug	COVID-19 Patients received Early EUA Drug	COVID-19 Patients received mAbs
Gender			
Male			
White	5.67% **	2.71% ***	2.34% ***
Black	6.02% ***	4.19% ***	1.54% ***
Asian	6.52% ***	4.88% ***	1.08% ***
Other Race	1.91% ***	1.58% ***	0.29% ***
Female			
White	5.06%	1.99% ***	2.34% ***
Black	6.03% ***	3.57% ***	1.99% ***
Asian	4.94%	3.16% ***	1.11% ***
Other Race	2.00% ***	1.47% ***	0.46% ***
Rural Urban Commuting Area			
Metropolitan			
White	5.65% ***	2.39% ***	2.55% ***
Black	5.97% ***	3.82% ***	1.79% ***
Asian	5.67% **	3.95% ***	1.10% ***
Other Race	2.10% ***	1.67% ***	0.39% ***
Micropolitan			
White	4.14% ***	1.89% ***	1.81%
Black	6.91% ***	3.76% ***	2.09% ***
Asian	4.11%	2.97% **	0.64% ***
Other Race	1.03% ***	0.53% ***	0.28% ***
Small Town/Rural			
White	4.14% *	2.25% ***	1.21%
Black	6.49% ***	4.01% ***	1.61% ***
Asian	5.61%	2.43%	1.87%
Other Race	0.95% ***	0.60% ***	0.21% ***
Multi-day Stay			
Yes			
White	9.39% ***	6.16% ***	2.71% ***
Black	10.99% ***	8.50% ***	2.23% ***
Asian	13.16% ***	11.04% ***	1.71% ***
Other Race	7.10% ***	6.07% ***	1.05% ***
No			
White	4.96%	2.20% ***	2.10% ***
Black	5.87% ***	3.73% ***	1.72% ***
Asian	5.80% ***	4.10% ***	1.07% ***
Other Race	1.80% ***	1.39% ***	0.35% ***

^*^p<0.1, **p<0.05, ***p<0.01

**Fig. 2:**
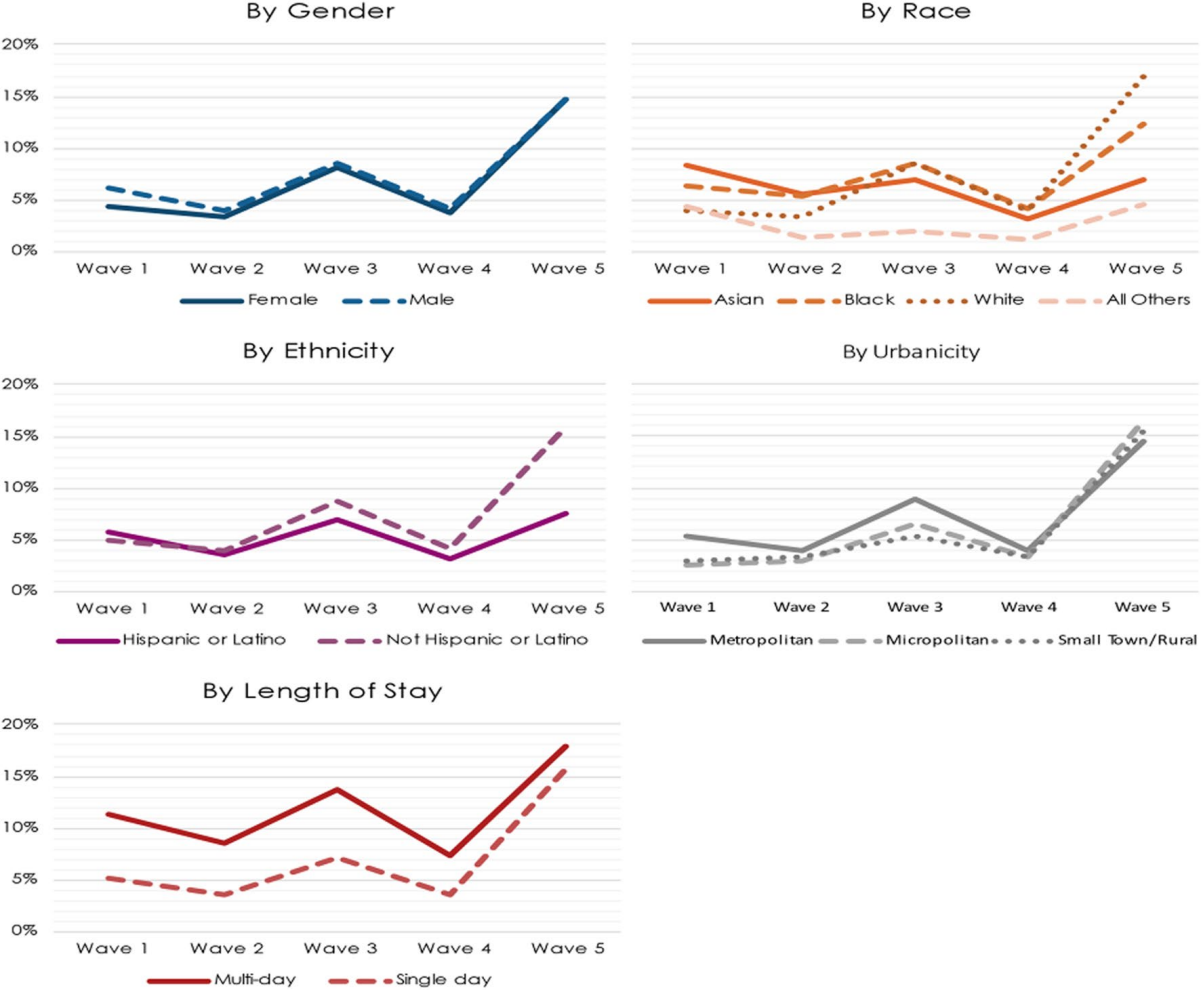
Mean Exposure to Any EUA Over Time. Note: Wave 1 is January 18, 2020 – September 9, 2020 (total number of patients=502,143); Wave 2 is September 10, 2020 – June 23, 2021 (total number of patients=1,624,667); Wave is June 24, 2021 – November 3, 2021 (total number of patients=685,050); Wave 4 is November 4, 2021 – March 23, 2022 (total number of patients=1,363,351); Wave 5 is March 24, 2022 – September 7, 2022 (total number of patients=285,172)

Figure 2 shows how the mean rate of exposure to any EUA drug for COVID-19 changed over waves by demographic categories. The likelihood of exposure to any EUA drug for COVID-19 was greatest in Wave 5 and smallest in Waves 2 and 4. The likelihood of exposure by demographic groups appears to exhibit some variation over time. Early on in the pandemic, Female patients were less likely to receive EUA drugs for COVID-19 than Male patients, and Black and Asian patients were more likely to receive EUA drugs than White patients. Beginning in Wave 3, those patterns were changing. Female patients were almost equally as likely as Male patients to receive EUA drugs for COVID-19 in Wave 3, and the gap continued to narrow through Wave 5. Black and White patients were almost equally as likely to receive EUA drugs in Waves 3 and 4, with Asian patients becoming less likely than either Black and White patients. We see much wider differences in Wave 5, with White patients being more likely, and Asian patients being less likely, than Black patients to receive an EUA drug for COVID-19. Other Races were consistently among the least likely to receive an EUA drug throughout the pandemic.

The likelihood of receiving an EUA drug by ethnicity also shifted over time. Early on, Hispanic patients were slightly more likely than non-Hispanic patients to receive an EUA drug for COVID-19. In Wave 2, however, the relationship had shifted, with non-Hispanic patients slightly more likely to receive EUA drugs. The gap widened substantially in Wave 5. Patients in Metropolitan areas generally had a higher likelihood of exposure to EUA drugs for COVID-19 compared to patients in Micropolitan and Small Town/ Rural areas throughout the study period, except in Wave 5 where the relationship reversed. Lastly, patients that had a multi-day visit were more likely to be exposed to any EUA drug for COVID-19 than patients with single day visits throughout the study period, although the gap did narrow in Wave 5.

## Conclusions and Discussion

Equitable access to health care has been a long-standing goal in the United States and the COVID-19 pandemic has likely complicated those efforts [11]. By some measures, the pandemic appears to have reduced recent gains in equitable access to health care. As Tiperneni et al. (2022) find, despite public guidance on equitable COVID-19 vaccine distribution, socially disadvantaged regions tended to have higher rates of COVID-19 incidence and mortality [12]. Communities of color [13], women, and lower-income individuals faced greater odds of experiencing adverse outcomes associated with the pandemic, including medical care inaccessibility, delayed medical care [14] and cancer treatment [15], and mental health problems. On the other hand, expanded access to telehealth services during the pandemic may have been particularly beneficial to these groups [16,17].

To better understand the impact of COVID-19 on access to health care and to ensure equitable allocation of health care system resources for more vulnerable populations, studies on the prevalence of COVID-19 and utilization of treatments across various patient groups at the individual level are crucial. Our study uses individual-level data from a national COVID-19 cohort sample to identify the exposure of patients to EUA drugs authorized for COVID-19. By comparing mean rates of exposure to these EUA drugs across patient demographics throughout the pandemic, our study identifies differences in access to these drugs as well as how those differences have changed over time.

Our findings regarding the use of mAbs by race and geographic region are largely consistent with previous findings that the use of mAb treatments is less common in racial and ethnic minority groups [6,18]. One study, using data from a convenience sample of health care facilities, finds lower use of monoclonal antibody treatment among Black, Asian, Other Race, and Hispanic patients with positive SARS-CoV-2 test results relative to White and non-Hispanic patients [6]. Our results show that Black and Hispanic patients were more likely to receive early EUA drugs but lower exposure to mAb’s than Whites and non-Hispanics. The large racial and ethnic disparities in COVID-19 cases early in the pandemic could help to explain why Black and Hispanic patients experienced higher rates of EUA drug exposure at the start of the pandemic. During the winter and spring of 2020, Black and Hispanic individuals experienced a higher rate of COVID-19 cases than Whites, even after accounting for underlying medical conditions and geography. On the other hand, disparities in access to care and the social determinants of health may have influenced the likelihood of access to mAb therapy. Patients usually receive mAb therapy by intravenous infusion or subcutaneous injection by a health care provider and were generally accessed by physician referral. Patients who lacked existing relationships with healthcare providers, whether because of a lack of insurance and inability to pay, geographic distance, or social and community factors, may have found it more difficult to access mAb therapies. Indeed, research has found that insurance status, social vulnerability, and geography may have contributed to disparities in access to mAb therapy [19].

Our results also showed that Female patients experienced lower rates of exposure to any EUA drug and to early EUA drugs than Male patients, although over time, that gap lessened. We also find that the difference in exposure rates by length of stay lessened over time, perhaps due to the availability of monoclonal antibody therapies in outpatient settings. For most of our study period, patients in Metropolitan areas were most likely to receive EUA drugs for COVID-19, although in Wave 5 that was no longer the case. These findings are similar to what has been discussed for EUA COVID-19 vaccination use [20,21]. The narrowing of the gap in EUA drug exposure by gender, rural-urban classification, and length of stay over time is likely due to the reduced information gap and improved public messaging on the availability of EUA drugs for COVID-19. However, the differences by race and ethnicity have generally persisted, which highlights the need for effective strategies to further improve health equity in access to these important therapies.

This study is subject to some limitations. First, while the data we use come from one of the largest collections of COVID-19 patient data in the United States, socially disadvantaged and underserved groups with limited access to health care resources may be underrepresented. Given this undercounting of patients with COVID-19, our rates of exposure to COVID-19 EUA drugs for these population groups may not be representative. Second, we present rates of exposure without controlling for underlying health conditions or severity of disease (although we do present an analysis using length of stay as a proxy for disease severity). Given that EUA drugs authorized for COVID-19 often specify disease severity or risk (for example, remdesivir was authorized for patients with mild to moderate COVID-19 who were at high risk for severe disease), our analysis risks confounding observed demographic characteristics with unobserved health conditions. Thus, results should be read as a broad study on disparities in exposure to COVID-19 EUA non-vaccine treatments. We do not make claims as to the causes of the disparities presented. Future research incorporating a comprehensive set of controls for confounding factors is warranted to further our understanding of the disparities in the exposure to COVID-19 EUAs.

The COVID-19 public health emergency declared under the Public Health Service Act expired on May 11, 2023. However, existing EUAs for products will remain in effect, and FDA may continue to issue new EUAs going forward when criteria for issuance are met [22]. While many previous studies on COVID-19 have highlighted age- and sex-related differences in vaccine coverage and health outcomes, our study sheds light on the differences in use of COVID-19 EUA non-vaccine treatment across various patient groups over time using the largest open U.S. database of more than 6 million COVID-19-positive cases. We find that Black patients were more likely to receive early EUA drugs while White patients experienced more exposure to mAbs. Male patients were more likely to receive EUA drugs overall than Female patients. Patients in Metropolitan areas had higher rates of exposure to EUA drugs than patients in other regions. Additionally, differences in the rates of exposure to EUA drugs by gender, rural-urban classification, and length of stay decreased over time while differences by race and ethnicity have generally persisted. Future studies exploring how economic and social factors and underlying health conditions impact disease surveillance and access to health care among underserved populations with COVID-19 would improve our understanding of the continuous use of EUAs and epidemiology during public health emergencies. They would also help ensure targeted health education and equitable allocation of health care system resources for more vulnerable populations.

## Data Availability

The data used in this study are proprietary and not publicly available. The data is provided from the National COVID Cohort Collaborative (N3C), and requires an agreement to access, thus restrictions on their availability apply. Researchers interested in accessing the data should visit https://covid.cd2h.org/enclave/.

## Footnotes

1 The EUA for Veklury (remdesivir) was revoked when the drug received full FDA approval. We exclude the following EUA treatments from our analysis: 1) Fresenius Kabi Propoven 2% and Propofol‐Lipuro 1% because these drugs were granted EUAs for sedation rather than as a therapy for COVID-19; 2) REGIOCIT replacement solution and Fresenius Medical, multiFiltrate PRO System and multiBic/multiPlus Solutions because they were granted EUAs for continuous renal replacement therapy rather than as a therapy for COVID-19; and 3) COVID-19 convalescent plasma because it is classified as a procedure in the N3C data rather than as a drug. The full name for Paxlovid is Paxlovid (nirmatrelvir tablets and ritonavir tablets, co-packaged for oral use).
